# Treatment Options for Problematic Internet Use

**DOI:** 10.1192/j.eurpsy.2023.150

**Published:** 2023-07-19

**Authors:** B. Dell’Osso

**Affiliations:** ^1^Department of Mental Health, Department of Biomedical and Clinical Sciences Luigi Sacco; ^2^“Aldo Ravelli” Center for Neurotechnology and Brain Therapeutic, University of Milan, Milan, Italy; ^3^Department of Psychiatry and Behavioral Sciences, Bipolar Disorders Clinic, Stanford University, California, United States

## Abstract

**Abstract:**

Research investigating interventions for problematic usage of the Internet (PUI) remains at an early stage but is steadily developing. In terms of therapies assessed through randomized clinical trials, literature suggests that cognitive behavioral therapy may be the most effective intervention but definitive statements as to its benefits need more testing. In relation to pharmacological treatments for PUI, studies have largely examined the efficacy of agents such as antidepressants and stimulants with a potential therapeutic effect of escitalopram, bupropion, methylphenidate, and atomoxetine. Another emerging form of potentially useful treatment involves non-invasive neurostimulation techniques such as transcranial magnetic stimulation and transcranial direct current stimulation. These interventions are thought to mediate their effect in PUI via stimulation of cortical brain cells and modification of their related functions. In summary, although the limited available treatment evidence includes some promising findings, there is a need for higher-quality research to develop best practice guidelines and determine cost-effective options in PUI treatment. The presentation will provide a state-of the-art overview in the field of therapeutics for PUI.

**Disclosure of Interest:**

B. Dell’osso Grant / Research support from: LivaNova, Inc., Angelini, and Lundbeck

